# Ultrastructure Analysis by Cryo-Electron Tomography Revealed Mesosomes in the Gram-negative *Delftia acidovorans*

**DOI:** 10.1007/s00248-026-02698-2

**Published:** 2026-01-26

**Authors:** Ana C. Afonso, Jack Botting, Manuel Simões, Lúcia Simões, Jun Liu, Maria José Saavedra

**Affiliations:** 1 LEPABE, ALICE, Faculty of Engineering, University of Porto, Rua Dr. Roberto Frias, Porto, 4200-465 Portugal; 2 Department of Microbial Pathogenesis, Yale School of Medicine, New Haven, CT 06536 USA; 3 New Haven Microbial Sciences Institute, Yale University, West Haven, CT 06516 USA; 4 Department of Biology, University of Minho, Campus de Gualtar, Braga, 4710-057 Portugal; 5 Department of Veterinary Sciences, CITAB, University of Trás-os-Montes e Alto Douro, Vila Real, 5000-801 Portugal

**Keywords:** Cytoplasmatic invaginations, Cryo-electron tomography, *Delftia acidovorans*, Membrane projections, Ultrastructure

## Abstract

*Delftia acidovorans*, a Gram-negative bacterium commonly found in diverse environments, can occasionally cause infections in immunocompromised individuals. Despite its environmental prevalence and clinical relevance, there is a notable lack of studies on the cellular ultrastructure of *D. acidovorans*. Characterizing this aspect is essential for understanding the bacterium aggregation behavior, which significantly influences biofilm formation, environmental adaptability, and potential pathogenicity in clinical contexts. This study employs cryo-electron tomography to investigate the cellular ultrastructure of *Delftia acidovorans*. Our observations of *D. acidovorans* revealed a supercoiling pattern in flagellar filaments and diverse outer membrane projections. Our major finding was the observation of cytoplasmic membrane invaginations resembling mesosomes seen in Gram-positive bacteria, offering new insights into the cellular architecture and potential functions of these structures in Gram-negative bacteria. Together, these ultrastructural insights reveal adaptations potentially linked to environmental persistence and interspecies aggregation.

**Supplementary Information:**

The online version contains supplementary material available at 10.1007/s00248-026-02698-2.

## Introduction

*Delftia acidovorans*, a Gram-negative bacterium belonging to the Comamonadaceae family, has been identified in various environments, including soils and aquatic systems [1]. While often considered a low-virulence organism, it has increasingly been associated with opportunistic infections, particularly in immunocompromised patients. Clinical cases include bacteremia, pneumonia, and infections associated with medical devices or dialysis systems [2–5]. These observations raise concerns about its capacity to act as an emerging nosocomial pathogen. However, the structural basis for its environmental persistence and potential virulence remains largely unexplored. Additionally, its versatility and adaptability to different environmental conditions make it a good subject for study, particularly concerning bacterial aggregation and biofilm formation [1]. Recently, this species, namely the *D. acidovorans* 005P strain isolated from drinking water (DW), was found to be an auto and coaggregating strain [6]. Bacterial autoaggregation refers to the ability of bacterial cells to clump together or adhere to each other, forming aggregates or clusters. Bacterial coaggregation, on the other hand, occurs when different bacterial species or strains come together to form mixed aggregates [7, 8]. Even though aggregates are transversal to different environments, including the atmosphere, aquatic environments (marine and freshwater), soil, and the intestinal microbiota of different living beings [9], not all bacterial species have this ability. Microbial aggregates manifest, for example, in various infections, with non-attached aggregates - multicellular clusters that form independently of a surface and remain suspended within the surrounding fluid or tissue environment - prevalent in chronic conditions associated with cystic fibrosis, chronic wounds, otitis media, and chronic osteomyelitis [10]. On the other hand, bacterial aggregates demonstrate remarkable biotechnological applications, such as biofilm-based wastewater treatment and enhanced bioremediation of environmental pollutants [7]. Despite their importance, the mechanisms governing bacterial aggregation remain poorly understood, especially in non-model organisms. Filling this knowledge gap is particularly urgent given growing evidence of the prevalence and ecological significance of unattached aggregates in both natural and engineered environments [7, 11]. For that, a deeper understanding of the organisms themselves, including their ultrastructural features, is a necessary first step.

Cryo-electron tomography (Cryo-ET) stands as a potent three-dimensional (3-D) imaging method, capable of observing molecular-level details of cellular components [12]. Its major advantage over other high-resolution imaging techniques is its applicability to frozen-hydrated whole cells, which allows for imaging of in-situ cellular components in their native contexts. It is thus ideal for unbiased characterization of relatively unstudied bacterial species like *D. acidovorans*.

In recent years, cryo-ET has enabled major advances in our understanding of bacterial morphology, offering unprecedented insights into the architecture of key cellular structures such as surface layers, outer membranes, and motility systems, many of which are directly linked to bacterial function, adaptability, and pathogenicity. For instance, Sogues et al. [13] revealed by cryo-ET the structural details of the Sap S-layer of *Bacillus anthracis*, a key virulence factor composed of the Sap protein. They obtained a high-resolution map of the S-layer, revealing conformational changes in the Sap monomers and providing insights into their assembly into a lattice. The study showed that the lattice maintains P2 symmetry with cross-like and ridge-like structures, although the exact arrangement of domains within the lattice could not be fully resolved. Sexton et al. [14] used cryo-ET to reveal the unique envelope architecture of *Thermotoga maritima*, showing that its outer membrane, the toga, is composed of extended sheaths of β-barrel trimers interspersed with small lipid bilayer patches. Their findings uncovered a bipartite outer membrane-tethering system involving surface layer homology-domain-containing proteins and membrane-spanning lipids, shedding light on the evolutionary transition between monoderm and diderm bacteria. Before that, Raddi et al. [15] systematically compared cellular structures from pathogenic *Leptospira interrogans* and saprophytic *Leptospira biflexa* using cryo-ET. Their study revealed structural differences in the cell envelopes of these two species, particularly in the lipopolysaccharide layer, which was crucial in understanding the biology and pathogenesis of *L. interrogans*.

Cryo-ET has also been widely used to study motility systems. While flagellar motors of microorganisms such as *Escherichia coli* and *Salmonella enterica* have been extensively studied, other motors, including more complex ones, have recently begun to be investigated. A recent study by Tan et al. [16] provided high-resolution cryo-ET structures of the *Salmonella Typhimurium* flagellar motor in both clockwise and counterclockwise rotational states, revealing dynamic conformational rearrangements of the C-ring and stator relocalization during switching. In *Borrelia burgdorferi*, cryo-ET revealed that the FliL protein assembles into a ring structure encircling the MotA/MotB stator complex. This ring enhances motor function by stabilizing the stator in its active conformation and facilitating its assembly around the motor, thereby optimizing torque generation essential for motility. These findings underscore FliL’s critical role in regulating stator dynamics and motor performance in spirochetes [17].​ Moreover, studies on *Pseudomonas aeruginosa* revealed unique features of its polar flagellar motor, such as prominent densities near the P- and L-rings, possibly linked to environmental adaptability [18].

Despite these advances, most cryo-ET studies focus on well-established model organisms. Environmentally relevant, non-model bacteria like *D. acidovorans* remain underexplored. However, a recent cryo-ET study by Afonso et al. [19] identified strain-specific differences in pili architecture between aggregating and non-aggregating *D. acidovorans* strains. The aggregating strain (005P) exhibited significantly shorter pili (≈ 25 nm) compared to the non-aggregating strain (009P) (≈ 223 nm), along with distinct spatial distributions. These observations suggested a link between pilus morphology and aggregation behavior.

Building on this initial observation, the present study aims to bridge the knowledge gap by delivering a comprehensive cryo-ET characterization of two *D. acidovorans* strains. By systematically describing their ultrastructural features, including cell envelope organization, flagellar architecture, outer-membrane projections and membrane invaginations, this work provides the first high-resolution structural overview of this environmentally and clinically relevant species. Moreover, this study establishes a structural baseline that will enable future investigations into how specific cellular features may contribute to ecological behavior, aggregation ability, biofilm development, and interactions with other microorganisms.

## Materials and Methods

### Bacteria Culture Preparation

Two *D. acidovorans* strains isolated from DW in the northern of Portugal were used [20]. Both strains are available in the publicly accessible culture collection at Micoteca da Universidade do Minho (MUM, https://www.micoteca.deb.uminho.pt/collection) in Braga, Portugal, with accession numbers MUM 24.11 (*D. acidovorans* 005P) and MUM 24.12 (*D. acidovorans* 009P). Bacteria were grown in R2A agar (Millipore, Sigma, GER), at room temperature (23 °C ± 2) overnight.

### Cryo-ET Data Collection

Cryo-ET imaging was performed on three independent cultures for each strain, and multiple tomograms were acquired from grids prepared from each culture. For that, bacteria from the agar medium were resuspended in phosphate buffer saline (PBS) until an optical density of 1 at 600 nm. Then, 10 nm of BSA gold tracers (Aurion, Wageningen, NL) were added to the bacterial suspensions. The mixtures were deposited on freshly glow-discharged cryo-EM grids (Quantifoil R2/1, Cu 200, Ted Pella, Inc., Redding, CA, USA) and then rapidly frozen in liquid ethane using a gravity-driven plunger apparatus as previously described [21].

The resulting frozen-hydrated specimens were visualized in a 300 kV Titan Krios electron microscope (Thermo Fisher Scientific, Waltham, MA, USA) equipped with a K3 summit direct detection camera and a BioQuantum energy filter (both Gatan, Pleasanton, CA, USA). Tilt series were acquired at 19,500x magnification (corresponding to a pixel size of 2.56) using SerialEM (RRID: SCR_017293) [22] and FastTomo script [22] based on a dose-symmetric scheme at defocus ∼4.5 μm. The stage was tilted from − 48° to + 48° at 3° increments. The total accumulative dose for each tilt series was ∼60 e^−^/Å^2^.

### Cryo-ET Data Alignment and Reconstruction

The frames of each tilt series micrograph were aligned in MotionCor2 (RRID: SCR_016499) [23]. Gold tracer beads were tracked to align all image stacks using IMOD (RRID: SCR_003297) [24]. Gctf (RRID: SCR_016500) was used for all aligned stacks to estimate defocus [25], and then the ctfphaseflip function in IMOD was used for contrast transfer function (CTF) correction. Alignment of the tilt series and tomographic reconstructions was performed in Etomo, which is part of the IMOD package [24]. Tomograms were reconstructed via the weighted back-projection and simultaneous iterative reconstruction technique and were 4×-binned for subsequent analysis [26]. Tomographic reconstructions were visualized using IMOD software [24]. Tomogram images were linearly contrast-adjusted across the full field to enhance visualization. All tomograms used in this study were newly acquired for this work - no datasets from previous publications were reused.

## Results

### Ultrastructure of the Cell Envelope

**Fig. 1:**
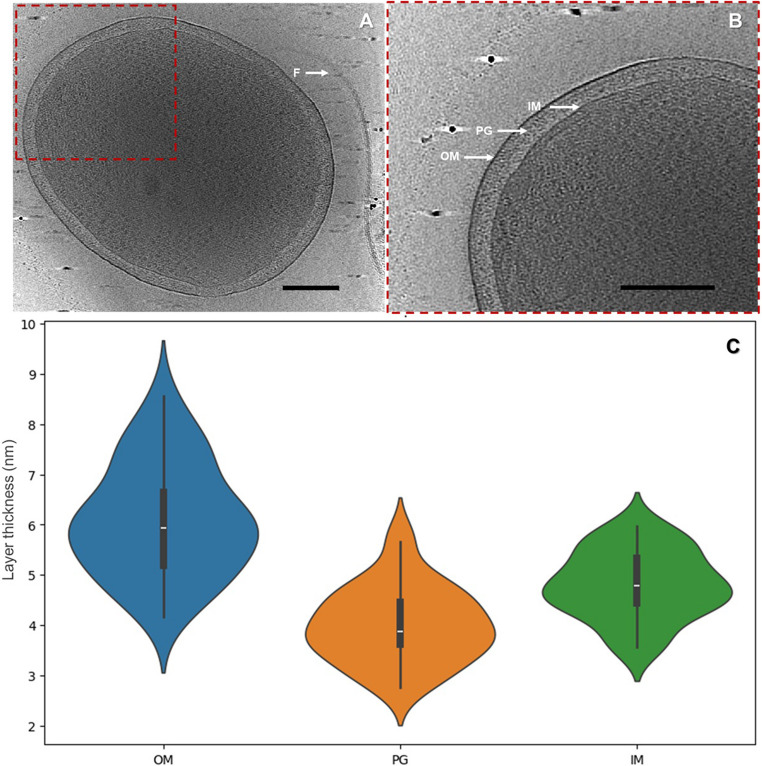
Ultrastructure from 3-D reconstruction of *D. acidovorans*. The flagellum (F), outer membrane (OM), peptidoglycan layer (PG) and inner membrane (IM), can be seen from the picture. (**A**) corresponds to the cryo-ET slice of a *D. acidovorans* cell. (**B**) is an enlarged view of the boxed region in (A), revealing the structural details of the cell envelope. Panel (**C**) shows the distribution of widths for each layer of the cell envelope in the form of violin graphs. Google Colab, an online platform that allows for the accomplishment of Python code in a web-based environment, was used to create violin plots using the Seaborn and Matplotlib libraries. The scale bar is 200 nm

Cryo-ET of intact *D. acidovorans* cells provided a detailed view of cell morphology and envelope (Fig. 1). *D. acidovorans* has a rod shape, which can be more or less elongated, and a double membrane architecture. The cell envelope is composed of an outer membrane (OM), peptidoglycan layer (PG), and inner membrane (IM) (Fig. 1B). OM proteins are the major component of the outer leaflet of the OM since LPS was not evident. No differences were observed between strains for the envelope layers (Figure S1). Envelope thickness measurements were obtained from a total of 33 cells, using multiple measurement sites per cell at regions where the membrane layers were clearly resolved. Overall, OM was always the thickest (6.10 ± 1.11 nm), followed by IM (4.81 ± 0.67 nm) and PG (4.01 ± 0.75 nm) (Fig. 1C).

### Flagellar Organization

**Fig. 2:**
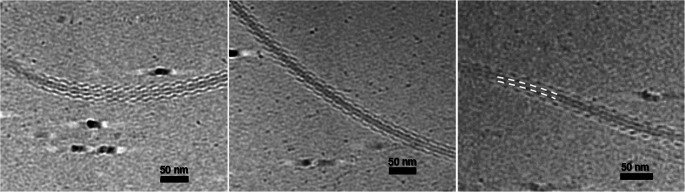
3D reconstructions of the *D. acidovorans* flagellar filament showing its supercoiled architecture. An external electron-dense layer outlining the central protofilament bundle is visible (white dashed line), corresponding to the exposed outer domains of the flagellin subunits. The scale bar is 50 nm

Both strains exhibited flagella (Figs. 1A and S1), with striking supercoiled conformations (Fig. 2). However, the number and position of the flagellum can vary between cells, being monotrichous or amphitrichous, and varying between a lateral or polar position (Figure S2). Positioned within the supercoil curve, the shorter protofilaments are found on the inner side, whereas the longer protofilaments are situated on the outer side (Fig. 2). Additionally, visible around the filament, an external density surrounds the central protofilament bundle (Fig. 2), corresponding to the outer domains of the flagellin subunits.

**Fig. 3:**
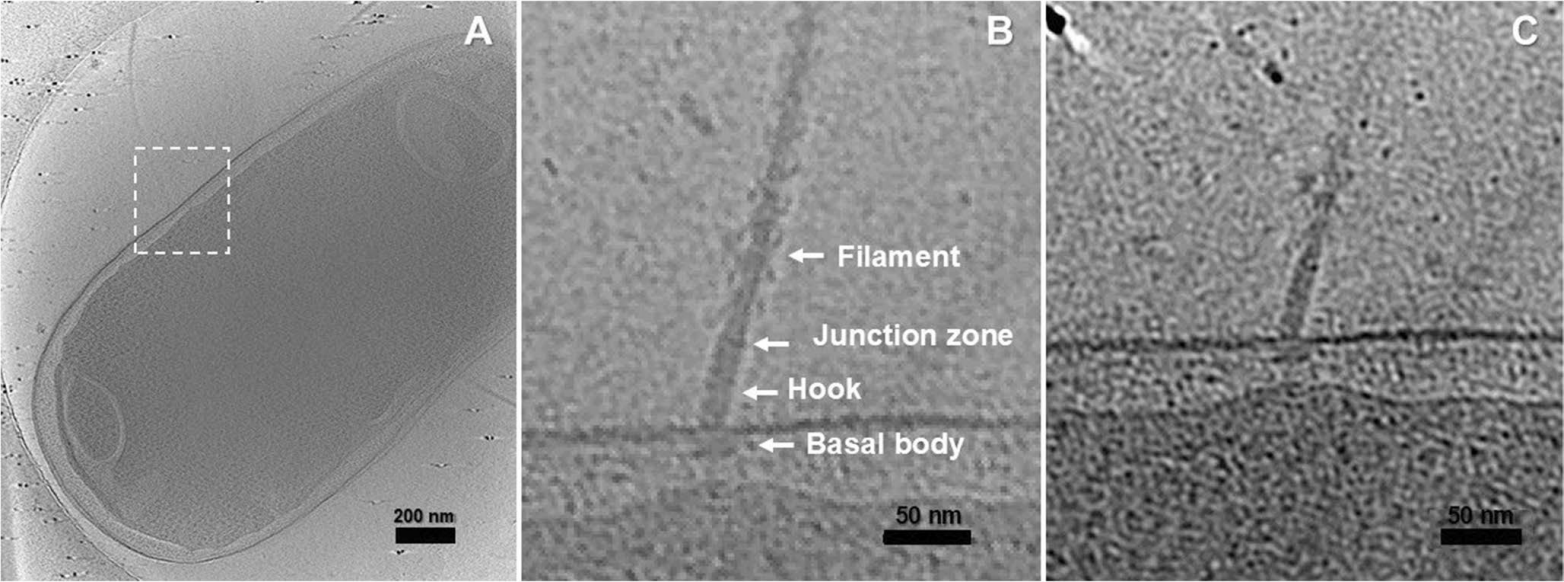
Ultrastructure from 3-D reconstruction of flagellum motor. (**A**) corresponds to the cryo-ET slice of a *D. acidovorans* cell. (**B**) and (**C**) are an enlarged view of the boxed region in (A)

The architecture of the flagellar apparatus in *D. acidovorans* was also visualized. In Fig. 3B (and Movie 1), the components comprising the flagellum are notably displayed, including the basal or motor body, the hook, the junction zone, and the filament, from the innermost to the outermost regions of the cell. The basal body is located between the IM and OM. In fact, periplasmic embellishments to the core motor structure are visible (Fig. 3C). Regarding the hook (≈ 49 nm tall and ≈ 13 nm diameter), it emerges from the upper part of the basal body located in the OM and separates from the filament through the junction zone (≈ 8 nm tall).

### Outer Membrane Projections

**Fig. 4:**
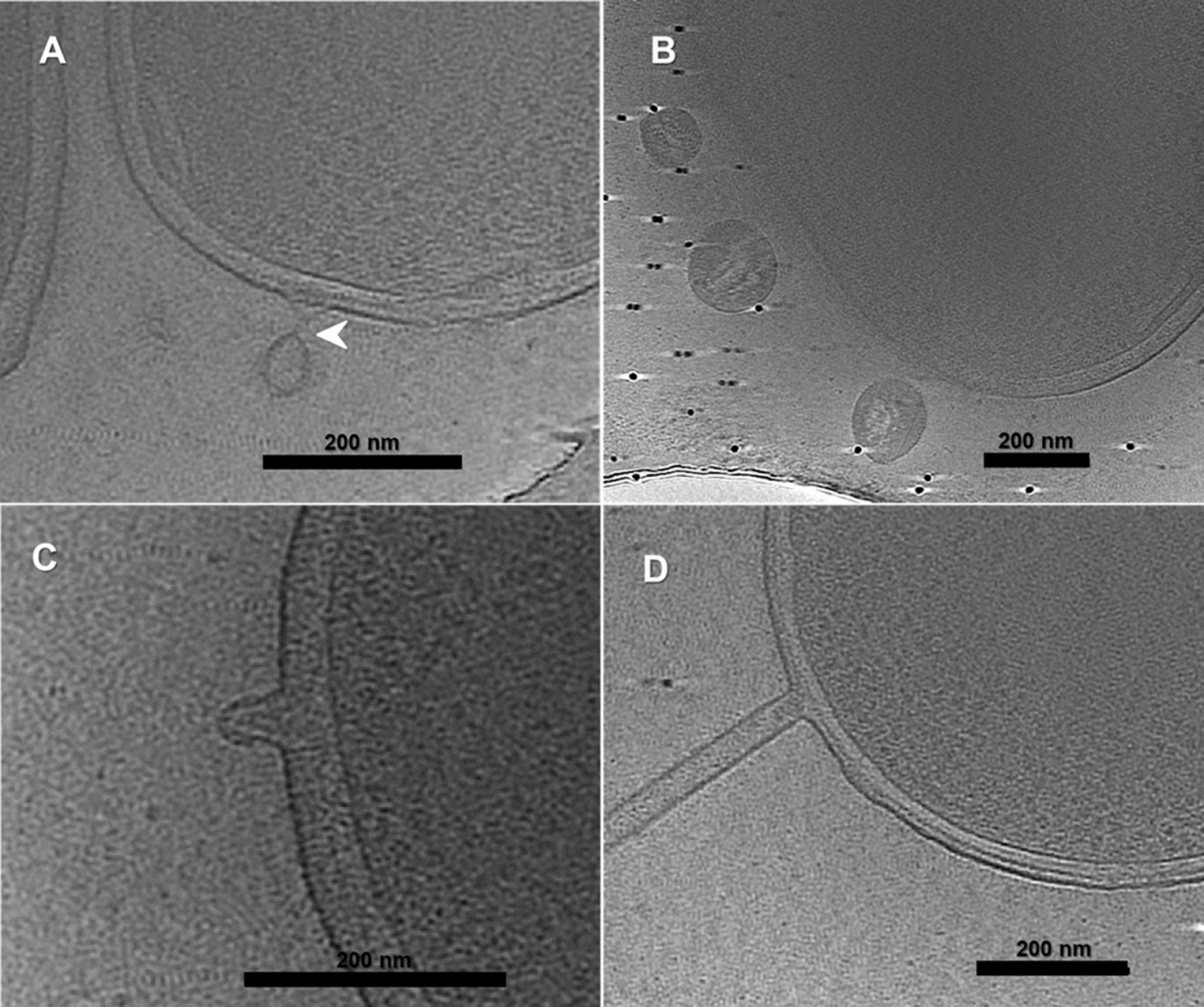
3D reconstructions from cryo-ET showing different types of outer membrane projections. (**A**) Budding and (**B**) detached outer membrane vesicles (OMVs); (**C**) protein complexes on outer membrane extensions (OMEs); (**D**) Tubes with a uniform diameter and no clear internal scaffold. The arrowhead points to a narrow tubular structure of the outer membrane. The scale bar is 200 nm

Three distinct types of OM projections, including budding and detached OM vesicles (OMVs) (Fig. 4A and B), protein complexes on OM extensions (OMEs) (Fig. 4C), and tube-like formations (Fig. 4D, Movie 2), were observed. These projections are frequently generated by the OM independently, without clear participation of the PG/IM layer. OM-derived projections were detected in approximately 13% of the cells examined, occurring in tomograms from both strains with comparable frequency and morphology.

Budding vesicles remained attached to the membrane (Fig. 4A), whereas detached vesicles were found in proximity to the cell (Fig. 4B). The vesicles displayed a range in diameter, averaging 140 ± 28 nm. Furthermore, OMVs were formed at bacterial cell division sites (Figure S3). Cryo-ET imaging of OMV formation (Fig. 4A arrowhead and Figure S3 arrowhead) demonstrated that these large vesicles budded off from septa through a narrow tubular outer membrane structure. Slices through cryo-ET of *D. acidovorans* showed the presence of protein complexes on OMEs (Fig. 4C). These complexes are approximately 50 ± 4 nm tall with a pointed top and a base approximately 44 ± 2 nm wide in the membrane. Another type of OMEs observed was in the form of a tube (Fig. 4D). These tubes had a uniform diameter of 37 ± 3 nm and 415 ± 148 nm in length.

### Cytoplasmatic Membrane Invaginations

**Fig. 5:**
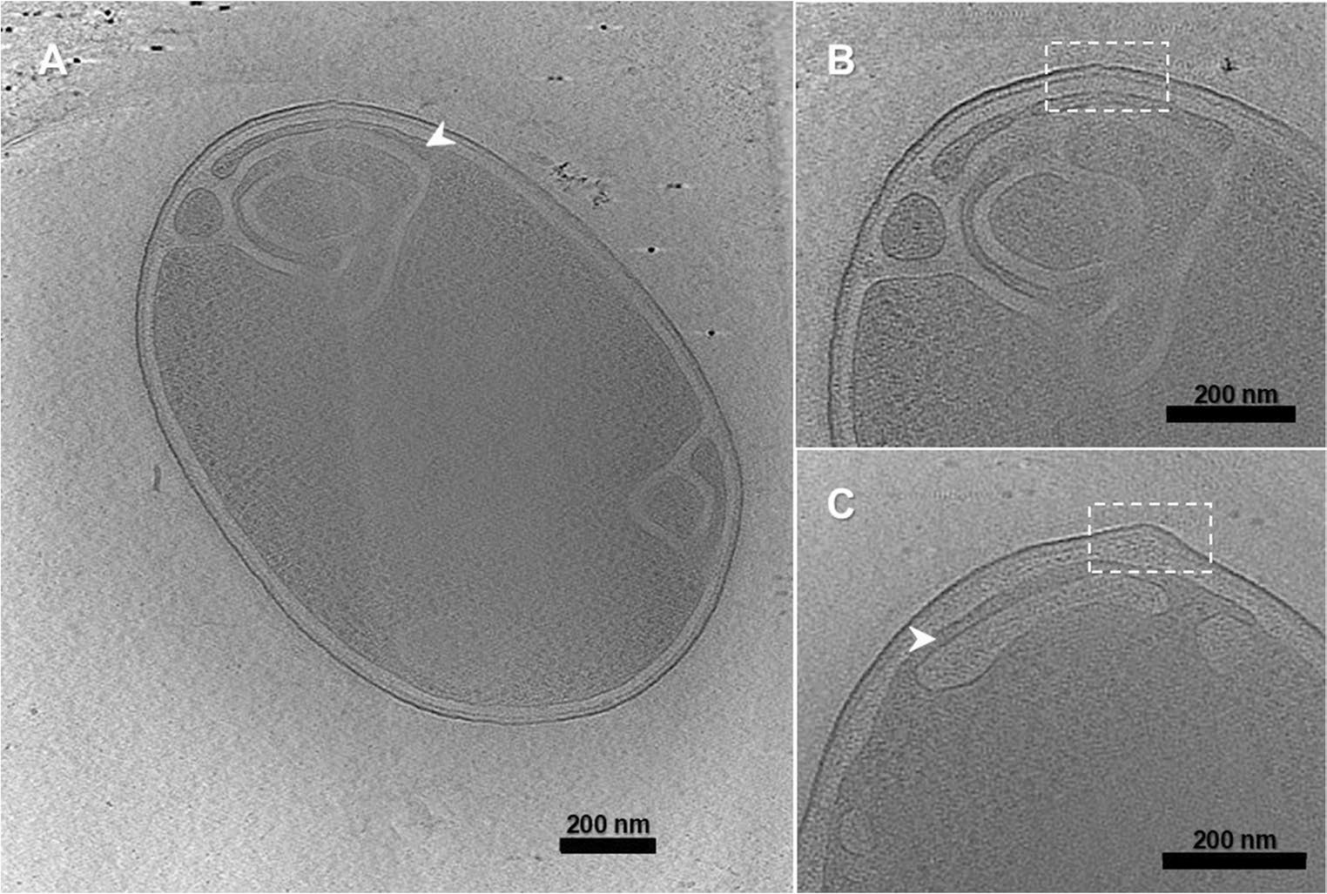
Cryo-ET 3D reconstructions showing cytoplasmic membrane invaginations. Invaginations were most observed at the cell poles, but occasional non-polar examples were also detected (Fig. 5A shows both cases). Panel **B** provides a magnified view of the boxed region in A and highlights a slight deformation of the cell envelope, which is similarly observed in panel **C**. The scale bar is 200 nm

Fifty-six cryo-ET tomograms revealed that the cytoplasmic membrane can form multiple invaginations, resulting in the enlargement of the periplasmic space (Fig. 5). The invaginations formed connections in depth, linking structures either above or below along the z-axis (Movie 3). Occasionally, these connections were established through small cytoplasmic ‘bridges’, no more than 40 nm in diameter (Fig. 5A and C). Other interesting findings were the fact that these invaginations are mostly located at the tips of the cells and have a curved shape, although less frequent non-polar examples were also observed. There did not seem to be a correlation between the formation of invaginations and the formation of outer membrane structures, as these occurred in different regions of the cells, as shown in Fig. 4. However, in two tomograms, an irregularity in the cell envelope was observed, which might be the origin of mesosome formation (Fig. 5B and C).

## Discussion

To the best of our knowledge, cryo-ET characterization of species within the Comamonadaceae family has not been previously reported. However, *Burkholderia* species, belonging to the same phylogenetic order, Burkholderiales, as *Delftia acidovorans*, have been well characterized using cryo-ET [27]. To provide a broader evolutionary and functional context for the features observed in *D. acidovorans*, we compiled a comparative overview of cryo-ET studies reporting similar ultrastructural elements. Table 1 summarizes cell envelope architectures, flagellar organization, outer membrane projections, and cytoplasmic membrane invaginations described in representative taxa. This comparison contextualizes the structural findings in *D. acidovorans* and highlights recurring and divergent patterns among different bacterial lineages.

**Table 1:** Comparative overview of bacterial ultrastructures observed through cryo-ET. The selection reflects studies referenced in the discussion section and is intended to provide broader evolutionary and structural context for the observations in *D. acidovorans*. “Not reported” indicate features not covered in the cited references

Organism	Phylogenetic group	Cell envelope	Flagellar architecture	OM projections	Cytoplasmic invaginations	Methodology	Reference
*D. acidovorans*	Betaproteobacteria	Gram-negative; OM > IM > PG; no evident LPS layer	Supercoiled filaments, variable polarity; sheath-like structure; conserved basal body	Budding OMVs, protein-decorated OMEs, tubular OM projections	Frequent invaginations with curved ends and interconnections; resemble mesosomes	Cryo-ET (Titan Krios, ~ 3–5 nm)	This study
*B. anthracis*	Firmicutes (monoderm)	Gram-positive; thick PG layer, S-layer lattice (Sap protein)	Not reported	Not reported	Not reported	Cryo-ET + subtomogram averaging (~ 7 Å)	[13]
*T. maritima*	Thermotogae	Gram-negative; bipartite OM (‘toga’): β-barrel trimer sheaths, lipid bilayer patches	Not reported	Not reported	Not reported	Cryo-ET (~ 4–6 nm)	[14]
*E. coli*	Gammaproteobacteria	Gram-negative	Supercoiled flagellar filament; conserved hook-basal body architecture	Not reported	Not reported	Cryo-ET; subtomogram averaging; structural fitting	[36]
*L. interrogans / L. biflexa*	Spirochaetes	Gram-negative; LPS-rich OM in pathogenic strain; envelope asymmetries	Internal flagella in periplasm	Not reported	Not reported	Cryo-ET (~ 3–5 nm)	[15]
*B. burgdorferi*	Spirochaetes	Gram-negative	FliL ring stabilizing stator complex	Not reported	Not reported	Cryo-ET + structural fitting	[17]
*P. aeruginosa*	Gammaproteobacteria	Gram-negative	Additional densities near P/L rings; possible adaptation	Not reported	Not reported	Cryo-ET (~ 4–5 nm)	[64]
*P. limnophila*	Planctomycetes	Gram-negative; double membrane system	Not reported	Not reported	Extensive cytoplasmic membrane system, interconnected	Cryo-ET (~ 3–5 nm)	[63]
*G. sulfurreducens*	Deltaproteobacteria	Gram-negative	Not reported	Not reported	Invaginations linked to increased metabolic flux under iron-limiting conditions	Cryo-ET (~ 4 nm)	[61]
*M. xanthus*	Deltaproteobacteria	Gram-negative	Gliding motility (non-flagellar)	Tube-like OM projections enabling intercellular exchange	Not reported	Cryo-ET + live imaging	[54, 56]
*B. pseudomallei*	Betaproteobacteria	Gram-negative	Periplasmic flagella (complex motor)	OMVs and surface structures	Not reported	Cryo-ET (Titan Krios, 300 kV); vitrified cells; FIB-milled lamellae; ~3–5 nm	[65]
*F. johnsoniae*	Bacteroidetes	Gram-negative	Gliding motility	Tube-like OM projections	Not reported	Cryo-ET (Titan Krios, 300 kV); OMEs with protein complexes; ~3–5 nm	[45]
*H. gracilis*	Betaproteobacteria (Comamonadaceae)	Gram-negative	Flagellar motor with periplasmic embellishments	Not reported	Not reported	Cryo-ET (Titan Krios, 300 kV); vitrified cells; in situ motor imaging; ~3–5 nm	[40]
*R. sphaeroides*	Alphaproteobacteria	Gram-negative	Not reported	Not reported	IM invaginations (photosynthetic)	Cryo-ET (Tecnai F20, 200 kV); light-induced membrane invaginations; ~4–6 nm	[62]

The overall cellular architecture observed in *D. acidovorans* is consistent with other Gram-negative bacteria, revealing well-defined IM and OM, a periplasmic space, and cytoplasmic compartmentalization. The OM was consistently the thickest of the three layers. This observation aligns with its dense protein composition, as previously described for other Gram-negative species [28]. The absence of evident LPS structures further supports the dominance of outer membrane proteins in this bacterium. In contrast, the thickness of the IM and PG layers differed only minimally and fell within the standard deviation. This suggests that the two layers are comparable in thickness under the examined conditions. Such similarity may reflect the structural compactness of the *D. acidovorans* envelope but also highlights the need for caution when interpreting fine-scale thickness measurements in cryo-ET, where biological variability and image resolution can influence layer distinction. Taken together, these observations indicate that although the OM stands out as the thickest layer, the cell envelope as a whole exhibits a relatively balanced architecture, without hypertrophy of any specific layer beyond what is typical for Gram-negative bacteria.

Cryo-ET has become an indispensable tool for studying the bacterial flagellum, providing a window into the nanoscopic world of this remarkable motility apparatus [29]. This technique has unveiled the intricate architecture of the filament, hook, and flagellar motor, offering unparalleled insights into their structural organization and interactions [29]. The detection of flagella corroborates previous phenotypic reports describing *D. acidovorans* as motile [30]. Cryo-ET revealed variations in the number and position of flagella, which may reflect physiological heterogeneity within the population or differing stages of the cell cycle. This variability is not uncommon among environmental bacteria, where motility often plays a critical role in colonization, biofilm formation, and nutrient foraging [31].

Extensive research has been conducted on the flagellar filament in numerous Gram-negative and Gram-positive bacteria [32, 33]. The filament is composed of a single flagellin protein, FliC [33]. This elongated homopolymeric set of flagellin protomers is organized in a helical arrangement similar to the structure of 11 hook protofilaments, and can assume two distinct conformations, denoted as L and R, resulting in alternative structures of left-handed and right-handed filaments [34]. In addition to driving translational movement in bacterial motility, the filament performs diverse functions including adhesion, pathogenicity, and immunomodulation [35, 36]. Cryo-ET revealed that the flagellar filament of *D. acidovorans* exhibits a supercoiled structure resembling that of *E. coli*, likely resulting from length differences among protofilaments [37]. As in *E. coli*, an external electron-dense layer is visible around the filaments` central domains, corresponding to the exposed outer domains of the flagellin subunits rather than to a membranous sheath [37]. These similarities suggest that core components of flagellar architecture are conserved across taxonomically distant species. However, despite this overall conservation, other environmental bacteria, such as *Caulobacter crescentus* and *Agrobacterium tumefaciens*, assemble filaments from multiple flagellins rather than a single FliC, reflecting the structural variability that exists even among species occupying similar environmental niches [38, 39]. Additionally, cryo-ET has provided detailed insights into the flagellar motor complex assembly [29]. The basal body, positioned between the inner and outer membranes, displays periplasmic embellishments similar to those observed in *Hylemonella gracilis*, another member of the Comamonadaceae family [40]. To resolve the in-situ motor structure in greater detail, subtomogram averaging will be required.

Membrane extensions (MEs) are observed in various bacteria and are especially well-studied in diderms, where they primarily originate from the OM and serve diverse functions [41–44]. The ability to produce OM projections was also identified for *D. acidovorans*. Our observations indicate that these projections are frequently generated by the OM independently, without clear participation of the PG/IM layer. This suggests a fluidic nature of the OM, which appears to be loosely associated with the cell body [15]. These projections exhibit a spectrum of structural diversity and functional roles. In the case of *D. acidovorans*, three distinct structures have been identified: budding and detached OM vesicles (OMVs) (Fig. 4A and B), protein complexes on OM extensions (OMEs) (Fig. 5C), and tube-like formations (Fig. 5D, Movie 2). The presence of these varied structures suggests diverse formation mechanisms and functions [45]. OMVs are ubiquitous vesicles derived from Gram-negative bacteria that encapsulate periplasmic content [41]. They are heterogeneous in size and composition, with their properties shaped by envelope structure, environmental conditions, and biogenesis pathways [41]. OMVs serve essential roles in bacterial communities, such as distributing enzymes for nutrient acquisition, recruiting iron, acting as decoys for bacteriophages or antibiotics, and facilitating DNA transfer [46–49]. While OMV research has largely focused on pathogens, their ecological roles in aquatic environments are increasingly acknowledged [46, 50]. In marine systems, OMVs are abundant and influence carbon fluxes [51], and similar roles are being proposed in freshwater environments. Notably, Silva et al. [52] provided ultrastructural evidence of OMV production by freshwater bacteria in situ, highlighting their active secretion into the environment. In *D. acidovorans*, OMV production has been linked to the degradation of phenanthrene, where vesicles and associated nanostructures (e.g., nanopods) enhance environmental interaction and metabolic efficiency [53]. OMEs, or outer membrane extensions enriched in protein complexes, have been described in several diderm bacteria, though their structures and functions remain poorly characterized. In a broad study of OMEs across species, Kaplan et al. [45] documented diverse forms, but none resembling the protein-decorated OMEs observed in *D. acidovorans*, suggesting a possibly unique assembly mechanism or function. Tube-like structures were also identified in our cryo-ET dataset. Similar formations have been described in *Flavobacterium johnsoniae* and *Myxococcus xanthus*, where they are involved in intercellular communication and the exchange of periplasmic or OM-bound molecules [45]. Previously, for *Myxococcus xanthus*, tubes displaying a uniform diameter and without a clear internal scaffold were observed and described as playing a pivotal role in mediating the intra-species exchange of periplasmic and outer OM-associated substances among cells, critical for the social dynamics characteristic of this species [54–56]. Taken together, these structures may play important ecological roles for *D. acidovorans*. OMVs could contribute to nutrient acquisition, detoxification, or DNA exchange in aquatic or soil environments, consistent with previous links between vesicle production and phenanthrene degradation in this species [57]. Protein-decorated OMEs may facilitate long-range interactions or the display of enzymatic complexes relevant to bioremediation, while tube-like projections could mediate cell–cell communication within multispecies biofilms. Although the precise functions of these projections in *D. acidovorans* remain to be elucidated, their diversity suggests a versatile membrane remodeling capacity with potential implications for signaling, biofilm development and environmental adaptation.

A key feature found in both *D. acidovorans* strains was the presence of elaborate invaginations of the cytoplasmic membrane into the cytoplasm, morphologically similar to mesosomes described in Gram-positive bacteria. Cytoplasmatic invaginations, also known as mesosomes, were once believed to be prevalent structures within bacterial cells, particularly among Gram-positive bacteria [58]. Initially attributed to roles in cell division, cell wall formation, and DNA replication [59], their prevalence and significance have been subject to debate. This debate largely arose from observations obtained using conventional electron microscopy, where chemical fixation, dehydration, and staining procedures were later shown to induce artifactual membrane structures [60]. However, the introduction of cryogenic preparation techniques, particularly cryofixation, has fundamentally altered this perspective by enabling preservation of cells in a fully hydrated, near-native state. Inner membrane convolutions have been described recently in the Gram-negative soil bacterium *Geobacter sulfurreducens*, where they were linked to an increase in metabolic flux, particularly under iron-limiting conditions [61]. Notably, these structures were observed using cryo-electron tomography and interpreted as biologically relevant membrane architectures. This finding was in line with intracytoplasmic membrane invaginations observed previously in *Rhodobacter sphaeroides* [62], further supporting the existence of organized inner membrane invaginations in Gram-negative bacteria. This correlation is intriguing, as *D. acidovorans* is known to inhabit environments with fluctuating iron availability, such as soil and aquatic systems where it participates in bioremediation and iron cycling. Iron is a critical cofactor for many bacterial enzymes and is often a limiting resource in microbial ecosystems. The formation of cytoplasmic invaginations in *D. acidovorans* may reflect an adaptive response to enhance surface area for specific processes, such as siderophore production, iron uptake, or metabolic flux under iron-limited conditions. Interestingly, the invaginations appeared to be interconnected across different cellular planes, suggesting a complex 3-D organization. Similarly, Boedeker et al. [63] used cryo-ET to investigate the Gram-negative *Planctopirus limnophila* cells, revealing a complex membrane organization with distinct invaginations connected in the z-direction. These connections were also occasionally formed by ‘bridges’ around 50 nm in diameter or by thin tube-like or disk-like invaginations of the cytoplasmic membrane [63]. The fact that these invaginations are located at the tips of the cells and have a curved shape, are consistent with Howley et al. [61] and Tucker et al. [62], respectively. Although the consistency of the observed invaginations is noteworthy, we critically evaluated the possibility that they might be artifacts of the sample preparation process. To further ensure the reliability of these observations, we systematically confirmed the presence of structural features across multiple tomograms, including datasets acquired from independent cultures of both *D. acidovorans* strains. The invaginations were observed in approximately 35% of the cells investigated, and their consistent morphology and location across different imaging sessions supports their biological relevance. Importantly, the use of cryo-fixation techniques, which bypass chemical fixation, minimizes the risk of preparation artifacts traditionally associated with mesosome-like structures. The similarity of our observations to those described in other cryo-ET studies (e.g., in *G. sulfurreducens* and *P. limnophila*) reinforces their authenticity and potential functional role in membrane dynamics.

## Conclusions

In conclusion, this study provides a comprehensive analysis of the cellular ultrastructure of *D. acidovorans*. Using cryo-ET, we were able to visualize and characterize key structural components, including the cell envelope, cytoplasmic invaginations, flagella, and OM projections. No ultrastructural differences were detected between the two strains in any of the features analyzed in this study, indicating that the cellular structures described here represent conserved traits of *D. acidovorans* species rather than strain-specific characteristics.

The detailed 3-D reconstructions revealed that both strains possess a rod-shaped morphology with a double membrane architecture, consistent with other Gram-negative bacteria. Our analysis of flagellum provided detailed insights into the supercoiling flagellar organization and motor structure, comparable to other well-studied bacterial species and potentially influencing motility and aggregation. We also identified different types of OM projections, such as budding and detached outer membrane vesicles, protein complexes on outer membrane extensions, and tube-like formations, underscores the versatility and adaptability of *D. acidovorans* in diverse environmental niches. Finally, the presence of mesosome-like structures in *D. acidovorans* highlights a broader relevance of cytoplasmic membrane dynamics in bacterial physiology, particularly in nutrient cycling and adaptation to environmental stresses. These findings, when linked to studies on *G. sulfurreducens* and other bacteria, suggest that membrane invaginations play a significant role in bacterial cell biology, potentially enhancing nutrient uptake, signaling, and compartmentalization.

Although this study did not include infection models, the ultrastructural features identified in *D. acidovorans* are suggestive of functions relevant to microbial pathogenicity. OMVs and membrane projections are known mediators of virulence factor secretion in pathogens. The complex flagellar structure may facilitate adhesion and motility within host tissues or biofilms, and cytoplasmic invaginations may optimize nutrient uptake under host-imposed stress. Future work should assess the contribution of these features to colonization, immune evasion, and resistance to antimicrobial treatments, particularly in healthcare-associated environments. This study demonstrates the power of cryo-ET as a robust tool for unbiased bacterial characterization, offering valuable insights into the ultrastructural features and functional implications of non-model organisms like *D. acidovorans*.

Ultimately, understanding the mechanisms underlying bacterial aggregation and cellular processes in *D. acidovorans* will contribute to our broader understanding of microbial ecology and evolution. The identification of membrane invaginations and surface projections opens avenues for functional exploration and highlights the value of high-resolution imaging in non-model organisms.

## Supplementary Information

Below is the link to the electronic supplementary material.

Supplementary Material 1 Movie 1. 3D tomographic reconstruction and overlay of the segmented structures showing the structures of the flagellum. Movie 2. 3D tomographic reconstruction and overlay of the segmented structures showing tube-like outer membrane structures. Movie 3. 3D tomographic reconstruction of the cell tomograms showing cytoplasmic invaginations and cytoplasmatic “bridges”.

Supplementary Material 2

## Data Availability

The data underlying this article are available in the article and in its online supplementary material.
