# Supplementary material for: Ultrastructure Analysis by Cryo-Electron Tomography Revealed Mesosomes in the Gram-negative Delftia acidovorans
Source: Microb Ecol. 2026 Jan 26;89:48. doi: 10.1007/s00248-026-02698-2 (PMC12901137; doi:10.1007/s00248-026-02698-2)
Supplement: Supplementary file 1 [file 248_2026_2698_MOESM2_ESM.docx]

**Supplementary Information**

**Ultrastructure analysis by cryo-electron tomography revealed mesosomes in the gram-negative *Delftia acidovorans***

**Figure S1.** Ultrastructure from 3-D reconstruction of *D. acidovorans*. The flagellum (F), outer membrane (OM), peptidoglycan layer (PG) and inner membrane (IM), can be seen from the picture. (A) corresponds to the cryo-ET slice of a *D. acidovorans* 005P cell. (B) corresponds to the cryo-ET slice of a *D. acidovorans* 009P cell. (B) and (D) are an enlarged view of (A) and (C), respectively, revealing the structural details of the cell envelope. The scale bars are 200 nm.

**Figure S2.** Montage showing the differences in the number (monotrichous or amphitrichous) and position (lateral or polar) of the flagellum for *D.* acidovorans cells. It can be monotrichous and polar (A); monotrichous and lateral (B); amphitrichous and polar (C); or amphitrichous and lateral (D). The scale bars are 200 nm.

**Figure S3.** 3-D reconstruction shows budding outer membrane vesicles (OMVs) formation at the bacterial cell division sites. The arrowhead points to a narrow tubular structure of the outer membrane. The scale bar is 150 nm.
